# Celiac disease - a pluripathological model in pediatric practice

**DOI:** 10.3389/fimmu.2024.1390755

**Published:** 2024-04-23

**Authors:** Vasile Valeriu Lupu, Maria Oana Sasaran, Elena Jechel, Iuliana Magdalena Starcea, Ileana Ioniuc, Adriana Mocanu, Solange Tamara Rosu, Valentin Munteanu, Alin Horatiu Nedelcu, Ciprian Danielescu, Delia Lidia Salaru, Anton Knieling, Ancuta Lupu

**Affiliations:** ^1^ Faculty of Medicine, “Grigore T. Popa” University of Medicine and Pharmacy, Iasi, Romania; ^2^ Faculty of Medicine, “George Emil Palade” University of Medicine, Pharmacy, Science and Technology, Targu Mures, Romania; ^3^ Faculty of Medical Bioengineering, “Grigore T. Popa” University of Medicine and Pharmacy, Iasi, Romania

**Keywords:** celiac disease, autoimmunity, child, atypical onset, associated pathologies, polymorphous clinical picture

## Abstract

Being defined as an autoimmune, chronic pathology, frequently encountered in any age group, but especially in pediatrics, celiac disease (also called gluten enteropathy), is gaining more and more ground in terms of diagnosis, but also interest in research. The data from the literature of the last decades attest the chameleonic way of its presentation, there may be both classic onset symptoms and atypical symptoms. Given the impact played by celiac disease, especially in the optimal growth and development of children, the current narrative review aims to highlight the atypical presentation methods, intended to guide the clinician towards the inclusion of the pathology in the differential diagnosis scheme. To these we add the summary presentation of the general data and therapeutic lines regarding the underlying condition and the existing comorbidities. In order to place the related information up to date, we performed a literature review of the recent articles published in international databases. We bring forward the current theories and approaches regarding both classic celiac disease and its atypical manifestations. Among these we note mainly constitutional, skin or mucous, bone, neuro-psychic, renal, reproductive injuries, but also disorders of biological constants and association with multiple autoimmunities. Knowing and correlating them with celiac disease is the key to optimal management of patients, thus reducing the subsequent burden of the disease.

## 1 Introduction

Representing almost a constant in pediatric cases of recent years, celiac disease (CD) is classified by specialists as being on a fine line between over- and under-diagnosis. The two variables have as a causal background the existence of atypical presentations of celiac disease (which turn out to be the same/more present than the classic form). Added to this is the tendency to diagnose exclusively on symptomatic criteria. In support of these two statements, we find the increase in recommendations for starting a gluten-free diet (GFD) (1). Trying to broadly define the disease, Holtmeier W. et al. notes the presence of objectified maldigestion and malabsorption in predisposed persons, in case of ingestion of gluten-based products (wheat, rye). The symptomatology is doubled by epigastric pain, abdominal flatulence, acceleration of intestinal transit, steatorrhea, weight loss, anemia, growth deficiency, osteo- articular, neurological diseases or infertility. In order to establish the diagnosis, digestive endoscopy with biopsy is performed, together with the dosage of anti-transglutaminase antibodies or, in particular cases, of other specific biomarkers. The histopathological examination shows the flattening of the jejunal mucosa and the infiltration with lymphoid cells (2, 3).

Although it represents the gold standard in the treatment of the condition, GFD seems not to be a universal “medicine”, Veeraraghavan G. et al. emphasizing the existence of cases of children with non-receptive celiac disease. This is more common among girls, beginning with the slowing down of intestinal transit and abdominal pain, which cannot be controlled by an exclusion diet for at least 6 months (4). Among the risks of starting a GFD, we note the increase in the prevalence of nutritional deficiencies such as iron, ferritin, vitamin B12, folic acid and zinc among subjects exposed to this lifestyle (5). Adherence to the regimen is another key point, observed in only ¾ of the evaluated subjects (with a peak of failure among teenagers, caused by the absence of symptoms when ingesting small amounts of gluten), increasing compared to a decade ago (6).

Regarding the management, the Society for the Study of Celiac Disease reports as challenging the follow-up of the histological recovery of the damaged tissues due to the inconsistency with the clinical and serological presentation of the patient. Also, the hypothesis of a phenotypic division of the condition according to the clinical and immune characteristics is launched, the knowledge of which can improve the diagnosis rate and therapeutic efficiency (7). Screening is also important in the risk population (children with affected first-degree relatives), the curve of affect depending on sex, age and the identification of HLA-DQ (2/8) forms specific to the pathology. Meijer C. et al. noted the development of celiac disease among 135 out of 944 children included in this risk group. In them, a peak incidence was observed around the age of 4 years, with an increased ratio among girls compared to boys and homozygotes for the leukocyte antigen DQ2 (8).

Considering the wide spread of the pathology at the global level, but also the strong systemic noise triggered by it, we consider it opportune to know and raise awareness of the importance of early detection and adequate treatment of celiac disease. In this sense, we propose to develop the atypical forms of presentation among children and adolescents, along with general aspects of pathogenesis, diagnosis and therapeutic management.

We will therefore outline a general framework of signs and symptoms that should orient the clinician towards CD, by summarizing the data present in the specialized literature, obtained by accessing international databases: PubMed, EBSCO, Scholar, EMBASE. The selectedarticles will present current data, valid from a statistical point of view. To these are added future perspectives in CD therapy, intended to widen the horizons of interest in research. The text is mainly focused on the pediatric population. Since the pediatric population represents a vulnerable population, whose study imposes extremely strict ethical limits, and in order not to lose sight of particularly important aspects for the referenced pathology, we have enriched the reading with data on the adult population where there were not enough testimonies related to children. We are referring here mainly to the pathophysiological aspects of celiac disease or its manifestations. The data were therefore presented for illustrative purposes, to avoid the main reading biases that may arise from the omission of the theoretical data that are the basis of the acquisition of practical notions. Where this happened, we specified, avoiding to record percentages or definite statistical data due to their inconclusiveness with those in pediatrics. To create our database, we used general and specific terms related to “celiac disease”, “diagnosis of celiac disease”, “forms of celiac disease”, “treatment of celiac disease” and a wide range of associated conditions. Finally, we reduced the risk of bias by including in the list of references both pro and con papers regarding the controversial information in the current scientific literature.

## 2 Epidemiology

Presenting two peaks of incidence (at 2 and 40 years respectively), depending on the onset of symptoms, CD is characterized by a real prevalence much higher than the estimated one. The average delay in diagnosis is approximately 4-10 years. Thus, it is recorded that, for each positively diagnosed patient, there are approximately 7-10 patients with a missed diagnosis (2, 9). Percentage, the prevalence is represented by 1% in the general population, 3.9% in the case of siblings of people with CD, 10-20% in the case of relatives and 75-80% for monozygotic twins. The gender ratio is 2:1 in favor of women. Although it is considered a frequent pathology among young children, research in recent years attests to the increase in the diagnosis rate among older children. This is partly due to the application of serological screening methods (IgA anti-tissue transglutaminase or endomysium antibodies) to those with atypical presenting symptoms or belonging to risk groups (9, 10). In adults, Llorente-Alonso MJ. et al. reports the high maintenance of CD cases among women (4:1 ratio in favor of men) (11). The diagnosis rate of CD is dependent on demographic indicators and ethnicity, presenting an increased density of cases in Europe, unlike in South America. The main theory underlying the demographic differences in the density of celiac disease cases mainly concerns the variability of the genetic factors involved in the pathogenesis [human leukocyte antigen (HLA) and non-HLA genes]. On a secondary level, current medical literature also talks about the impact of environmental factors (e.g., wheat consumption, age at gluten intake, gastrointestinal infections, use of proton pump inhibitors or antibiotics, caesarean section rate and particularities of the breastfeeding process). All these aspects will be detailed further, from the perspective of extraintestinal manifestations (9).

The prevalence of extra-intestinal symptoms brings together, in descending order of occurrence, abdominal pain, poor weight curve, iron deficiency anemia, short stature, chronic constipation, skin manifestations (eczema) and reduction of bone mineral density (12, 13).

Regarding the factors that can precipitate the occurrence of autoimmunity and celiac disease, Andrén Aronsson C. et al. reports an increase in its risk proportional to each gram of gluten consumed per day (14). Another variable is represented by the type of birth, a physiological process that can leave an impression on the future course of the child in the sense of increasing the incidence of certain diseases (respiratory tract infections, asthma, obesity, disorders on the autistic spectrum, attention deficits or delays in neuro-psychic development). This aspect is generally attributed to an incomplete development of the infantile microbiota among infants born by caesarean section. To this are added particular aspects of the first years of life. However, the data regarding the impact of caesarean section, breastfeeding or the age of introduction of gluten in the diet on the increased risk of CD are contradictory (9, 15–17). Regarding the time of introducing gluten into the diet (6 versus 12 months), Lionetti E. et al. have objectified an increase in the incidence of autoimmunity related to celiac disease and CD in the first group. However, the difference was not maintained after the age of 5 years. The selective deficiency of immunoglobulin A (IgA) and particular HLA variants thus remain the most important predictors of the condition. However, genome-wide studies have found non-HLA risk factors common to other immune-related diseases (e.g., type 1 diabetes, rheumatoid arthritis, ulcerative colitis, and Crohn’s disease) (18–20).

Similarly, a correlation was made between viral infections (e.g., rotavirus, adenovirus, enterovirus, hepatitis C or Epstein-Barr virus) and the development of CD, anticipating their possible involvement in modulating the individual response to gluten. This interrelation is possibly influenced by the existence of a sequence of 8-12 amino acids similar to the toxic gliadin fraction. Vaccination exerts protective effects among children with gluten intake before 6 months (9, 20).

In light of the recent Covid-19 pandemic, Cakir M. et al. demonstrates an increase in the diagnosis of CD individually or in association with diabetes in the period March 2020-June 2021, in contrast to the pre-pandemic period. In support of the positive correlation between the acute respiratory infection and the escalation of the CD incident, we find the positivity of the serological markers of passing through the disease in a percentage of 36.3% of the children diagnosed in the pandemic (21). In the opposite sense, Lionetti E. et al. report a similar prevalence of Covid-19 among CD subjects compared to the general population.

However, the development of more severe forms or burdened by complications is not noticeable in them (22). Similar findings in the reference period were also objectified in the case of other autoimmune pathologies, such as systemic lupus erythematosus, where there was a marked increase in the rate of relapse, diagnosis of new cases and even a decrease in therapeutic control in pre-existing cases (23).

## 3 Pathogenesis

The physio-pathogenic cascade of celiac disease originates, as we mentioned previously, in the triggering of the body’s immune response as a result of the ingestion of gluten-based foods, in genetically susceptible individuals. The most common forms of human leukocyte antigen (HLA) associated with celiac disease are HLA-DQ2, HLA-DQ8 and, less commonly, HLA-DQ7. Despite the high degree of detection in the group of patients with symptomatic celiac disease, it is recorded that up to 20% of the general population possess these genes without manifesting a characteristic clinical picture during life. Thus, following the interaction between these and the deaminated peptide fragments, resulting from hard-to- degrade prolamins (gliadin, hordein, secalin, zein) under the action of tissue transglutaminase 2 (tTG2), CD4 T lymphocytes are activated. Following this, we encounter an increase in pro- inflammatory cytokines and chemokines, among which we distinguish interferon-gamma, interleukin 15 (IL-15), IL-2, IL-21 and tumor necrosis factor. The consequences are the infiltration of inflammatory cells and the promotion of cytotoxic CD8 T cells. Thus, atrophy of the villi of the mucosa, hyperplasia of the crypts and dysfunction of the intestinal barrier appear. These changes entail the promotion of pathogenic bacteria, the increase of translocation capacity and intestinal permeability, pathognomonic characteristics for celiac disease. In parallel, we aim to increase the production of antibodies directed at the import of tTG2 and gluten, important diagnostic markers, by B cells. Recently, Akbulut EU. et al. discuss the involvement of the IL-6 polymorphism (-572G/C) in dictating the susceptibility to the development of CD (2, 3, 7, 20, 24, 25). In addition to this mechanism, other factors that modulate the predisposition to CD include the premature cessation of breastfeeding and the gender of the patient, although the risk ratio for sexes in children is 1:1. The two exert their negative effect by decreasing the body’s defense capabilities against exogenous infections, as well as by promoting a hormone-dependent pro-autoimmune status (3). Finally, we can also add the impact played by low birth weight, lack of *H. pylori* colonization, SARS-CoV-2 infection or smoking status (24). Focusing on *H. pylori* infection among patients with celiac disease, we notice that this is an intensely debated topic in the literature. Summarizing the significant data, we can conclude that the frequency of *H. pylori* infection is lower among CD patients compared to control groups, both for children and adults (26–28). Where the two entities coexist, it is noted that children show milder forms of enteropathy. Therefore, it has been hypothesized that *H. pylori* infection may confer some protection against the development of severe degrees of villous atrophy. The main pathophysiological mechanism of the protective effect exerted by *H. pylori* has been attributed to a potential modulation of gluten immunogenicity among genetically susceptible patients (29).

In addition to the multitude of physiological processes that compete for the appearance of various pathologies, medical studies currently place the relationship between the disruption of the human microbiota and the escalation of the predisposition or the risk of associated comorbidities. In this sense, the balance of microorganisms, a constantly changing bioactive system, appears correlated with various diseases of the main organs (brain, heart, intestine, kidneys, lung, skin). The way in which the two entities communicate and influence each other is based on the existence of axes that connect the intestine with them. Thus, we found disturbances of the microbial flora in various atopies (asthma, dermatitis, food allergies), autoimmunity (systemic lupus erythematosus, CD, diabetes, autoimmune thyroiditis), organic insufficiency (cardiac, renal), neurological, oncological or metabolic disorders. To these are added inflammatory conditions (e.g., pancreatitis), respiratory infections, irritable bowel, gastroenteritis, esophagitis, gastroesophageal reflux disease or diverticular disease, pathologies frequently encountered in medical practice (30–36). The oral and intestinal microbiota of patients with CD is strongly influenced by a variety of factors, starting from the impact of genetic determinants, the environment (antibiotics, infections) and even the gluten-free diet. Their accumulation determines a state of microbial dysbiosis that affects the ability to take up and integrate peptides from food, being still under research if this is one of the causes or the effect of CD. The main microorganisms involved in the digestion of gliadin at the oral level are *Rothia, Actinomyces, Neisseria* and *Streptococcus*, while in the intestines of patients with active CD, an increase in *Proteobacteria* (e.g., *Neisseria*) was found in parallel with a decrease in *Firmicutes* and *Actinobacteria*. Thus, the hypothesis was outlined that in the small and large intestines of patients there are bacterial genera that influence the digestion of gliadin (e.g., *Lactobacillus, Streptococcus, Staphylococcus, Clostridium, Bifidobacterium*), their proteolytic activity being dictated by the amount of gluten ingested (20, 37). In this sense, current research focuses on the possible impact played by *Flavobacterium meningosepticum*, a bacterial endopeptidase that appears to be able to digest proline-rich peptides. However, the complete prevention of gluten toxicity by enzyme therapy is still controversial (2).

The systemic consequences of introducing a diet based on the exclusion of gluten are also worth mentioning. In this situation we can encounter deficiencies in vitamins, minerals or dietary fibers (source of short-chain fatty acids). Diet with excess fat can also precipitate an escalation of the risk of cardiovascular, metabolic diseases or body weight (in the sense of overweight or obesity) in the case of patients who do not benefit from adequate nutritional counseling. To this is added the influence played by nutrients such as vitamin A, vitamin E, selenium, calcium, iron, magnesium, zinc, omega-3 fatty acids, phytoestrogens or flavanols in the regulation of T cells and cytokine production. This balance is considered vital in the evolution of patients suffering from autoimmunity (38, 39).

## 4 Description of classic celiac disease

Due to the chameleonic ways of presentation, the diagnosis of celiac disease must be based on a complex and well-established protocol, made up of the corroboration of clinical and paraclinical data. The ultimate goal of following such protocols is primarily to reduce the risks of practitioners missing the correct diagnosis. Added to this are the benefits of early diagnosis and management of the pathology, aspects that improve the quality of life and “disabilities” of patients. In the following, we draw general lines in the recognition and certification of celiac disease in children, and then we draw attention to the particular forms of presentation.

Therefore, CD diagnosis starts gradually, from non-invasive to invasive, the choice of method being made according to the individual risk of each patient. Biologically, serology includes endomysial antibody (EMA), tissue transglutaminase (tTG) and deamidated gliadin peptide (DGP) assays, high sensitivity and specificity tests. Given the increased frequency in the general population of haplotypes associated with CD, despite the weak correlation with overt disease, HLA typing is considered to have a negative predictive value rather than a positive one. More invasive but considered the gold standard in difficult diagnostic cases (IgA deficiency, discrepant serology and the initiation of a gluten-free diet prior to testing), we find endoscopy doubled by intestinal biopsy (40).

Depending on the way of presentation and the response to the initiation of supportive therapy, children may be at risk of manifesting CD or may present the silent, with negative serology, refractory or non-responsive form. Cases with villous involvement but with characteristic negative serology require differential diagnosis with parasitic infection with *Giardia lamblia*, immunodeficiency, autoimmune or drug-induced enteropathy/in association with human immunodeficiency virus, Crohn’s disease or intestinal lymphoma (2, 9). In support of these statements, Oliveira GN. et al. notes, following the analysis of 159 patients, a prevalence of the classic disease of 60%, while the non-classic form was found in 25% of children.

Regarding the subclinical or potential forms, their prevalence was estimated at 5, respectively 10% of the number of cases (41). Among these forms, we choose to detail refractory CD, defined as the persistence of clinical symptoms and histological changes, generally unaccompanied by the escalation of autoantibody titers, despite a GFD followed for at least 12 months. The importance of its awareness resides in the increased risk of association with malignancies (intestinal lymphoma with T cells). Its prevalence is approximately 5% of the total cases of CD, being subdivided into two forms. For an easier presentation of the two forms (I and II), we refer to the exhaustive descriptions of type I - normal intraepithelial lymphocytic phenotype and, respectively, type II - clonal intraepithelial lymphocytic phenotype. The latter can be detected by means of immunohistochemistry, polymerase chain reaction methods or, more recently, flow cytometry. The definition of the clonal phenotype includes the loss of the normal surface markers CD3, CD4 and CD8 with preserved expression of intracytoplasmic CD3 (CD3ϵ) in >50% of intraepithelial lymphocytes (on the sample analyzed by immunohistochemistry) or >20%–25% (on the sample analyzed by cytometry in flow), doubled by the detection of T cell receptor chains (γ or δ) clonal rearrangement by polymerase chain reaction. The prognosis of type I is much better compared to type II, showing a better clinical/histological response to steroids or other immunosuppressive or biological drugs and less potential for lymphoma-malignancy (3, 9, 42).

### 4.1 Clinical

In celiac disease, the clinical picture of the pathology is in the first phase dependent on the age of diagnosis, without showing a significant correlation with the degree of damage to the intestinal mucosa. It should be noted that this has registered a significant increase in the last decade. The pathognomonic sign of CD is diarrhea with steatorrhea which, in small children, is doubled by anorexia, vomiting, flatulence and abdominal discomfort. Subsequently, these progress towards growth retardation, severe malnutrition, nutritional deficiencies, anemia or delay in the onset of puberty. It is noted that with advancing age, the clinical picture may become non-specific/atypical (2, 9, 24, 43). In this situation, the intestinal histology damage is more pronounced than in the classic/asymptomatic forms of the disease (44). The presence of age-related disease patterns was also studied by Tanpowpong P. et al. They demonstrated, through their analysis of 411 children and adolescents diagnosed with CD by biopsy, that age-dependent variations were present more frequently in the case of classic, gastrointestinal symptoms. On the other hand, age did not represent a significant variable in the case of non- specific symptoms (45).

Given the inclusion of the condition in the field of gastrointestinal pathologies, its initial extra-intestinal manifestations are considered atypical. Found in a large percentage of cases, the most important to know are small stature, skin-mucosal manifestations, osteoarticular, dental or skin appendages (hair) damage, endocrinopathies, hematological or neuro-psychic disorders. To these is added an increased risk of developing autoimmunity up to 10 times compared to the general population (3, 9). Being the basic theme of this article, all these manifestations will be detailed later.

### 4.2 Serological

The course of serological diagnosis of CD is well established based on protocols. Currently, the recommendations indicate the beginning of investigations by dosing IgA anti-tTG antibodies by enzyme-linked immunosorbent assay (ELISA) or, rarely, by radioimmunoassay (RIA). Due to the incrimination of gluten as the main trigger in the induction of symptoms, as well as the use of GFD as the current therapeutic gold standard, the necessity of testing during a normal diet, which includes gluten, is understood. This must precede the time of testing by at least 6 weeks. If it is impossible to achieve this, a gluten challenge test can be used (3-7.5 g/day, for 14 days). For the purpose of appropriate interpretation, the investigations must be completed by the evaluation of the serum IgA titer, to avoid cases of superimposition of normal values of anti-tTG over a selective immune deficiency. In order to obtain a reliable diagnosis in the case of a selective immune deficiency of IgA, the values of IgG antibodies are measured (9, 24, 46). However, it is necessary to clearly distinguish the selective IgA deficiency from the partial one. The latter is defined as total IgA with more than 2 standard derivations below the age average. In this case tTG IgA showed sensitivity up to 100% (47). A special recommendation is advanced in the case of children younger than 2 years where the immunoglobulin G (IgG) DGP test must be performed, due to the low sensitivity of tTG in this age group (9, 40). Liver damage and inflammation of the small intestine can also interfere with serological test results, precipitating a possible false-positive result (3).

The interpretation of positive values of anti-tTG IgA antibodies is reported according to a limit that exceeds 10 times the normal value. Depending on this, the need to perform a biopsy for diagnostic purposes is decided. Once this level is exceeded, the damage can be confirmed exclusively based on clinical-serological criteria (with the mandatory inclusion of anti-EMA antibodies in the protocol) with/without HLA typing. On the other hand, the positivity of IgG antibodies in the case of IgA deficiency does not exclude the biopsy, regardless of their value. In conclusion, the usefulness of serological testing in evaluating adherence to therapy should not be omitted. Thus, the markers become negative gradually, starting with the first half year after the initiation of the GFD (9, 46, 48).

### 4.3 Histopathologic

Although changes such as mucosal fissures, nodular mucosa (mosaicism), visible vascularization of the submucosa, bulbar atrophy or reduction of Kerckring folds are specific to CD, up to a third of newly diagnosed patients present a “clean” endoscopic image. Thus, for certainty, a puncture-biopsy with histopathological examination is recommended. The target biopsy area is represented by the duodenum. This is done in four quadrants doubled by collecting a sample from the level of the bulb. The preference for a multilocular pattern of analysis resides in the uneven intestinal touch encountered in CD. The main aspects that must be followed when objectify the diagnosis include the height of the intestinal villi, the depth of the crypts and the number of intraepithelial lymphocytes per 100 enterocytes (46, 49, 50). The most faithful way of classifying the histological forms of CD is according to the Marsh classification, brought up to date in the form of Marsh-Oberhuber and divided into six levels. Another way of classifying the intestinal damage is represented by the division into two groups (A – non-atrophic and B – atrophic). While group A targets an isolated increase of intraepithelial lymphocytes, group B (subdivided into B1 and B2) analyzes the inversion of the ratio of intestinal villi/crypts (normally over 3/1). However, the histological changes must be differentiated from other pathologies with similar manifestations. Among these we list autoimmune or chronic inflammatory conditions, *Helicobacter pylori* and other gastrointestinal infections, use of non-steroidal anti-inflammatory drugs or proton pump inhibitors, hypogammaglobulinemia (3, 49). Regarding atypical forms, Semwal P. et al. they reiterate the increase in their prevalence with advancing age and the main forms of presentation. To this are added findings regarding the lower risk of objective damage with the help of upper digestive endoscopy/histopathological examination in atypical forms, contrasting with classic ones. At the same time, histologically normal samples were found in increased numbers in the non-classical forms (51).

### 4.4 Pitfalls in diagnosis

Being faced with a complex pathology, with a variety of forms of presentation, CD evaluation can present a challenge for the clinician. In addition to this, the uncertainty hangs over the contexts in which screening is recommended, but also regarding the usefulness of classifying the disease in asymptomatic cases (7). Crossroads in the diagnosis and management of CD can refer to situations such as the young age of the patient, the discrepancy between the serological values and the histopathological changes, the objectification of increased intraepithelial lymphocytes (>25 IELs/100 enterocytes) without altering the villous structure or following a GFD at the time of investigation (40). Some of these may be attributed to uneven involvement of the duodenal mucosa, low gluten intake, or inappropriate biopsy orientation (9). Also, false negative results other than those stated above may appear in the case of the use of corticosteroids or immunosuppressive medication (48). In this sense, we discuss the findings of Kav T. et al. regarding the utility of enteroscopy in diagnosis. They emphasize its usefulness in the case of discordant serology-histopathology, in identifying locations of interest for biopsy, but also in the long-term follow-up regarding possible complications. To increase the efficiency of the method, the benefit of adding immersion in water is brought to the fore (52). His findings were also supported by subsequent studies, which emphasized the importance of monitoring intestinal changes in patients with CD with the help of the video capsule. The main arguments were represented by the ease of automating the investigation and thereby eliminating the possibility of bias (53, 54). In agreement with what was stated previously, in order to eliminate any diagnostic error, the initiation of a regular regimen, which includes gluten, is called for. Other minimally invasive ways to assess intestinal integrity in CD are double-contrast enema, small bowel ultrasound, MR enteroclysis and CT enterography. These are not currently included in the international guidelines as diagnostic methods of CD. However, they are useful and mainly used in follow-up, in the detection of intraluminal, mural (e.g., inversion of the jejunoileal fold pattern, transient intussusceptions, assessment of small intestine motility), mesenteric (e.g., enlarged mesenteric lymph nodes, mesenteric vascular engorgement) abnormalities mesenteric and transient intussusceptions) and intraabdominal (e.g., intestinal dilatation, increase in the volume of the gallbladder, free abdominal fluid) specific to CD and its complications (55–59). Thus, CD can most likely be excluded in the context of maintaining negative serological and histopathological results, despite a gluten-containing diet for 6-12 months (49).

**Figure 1:**
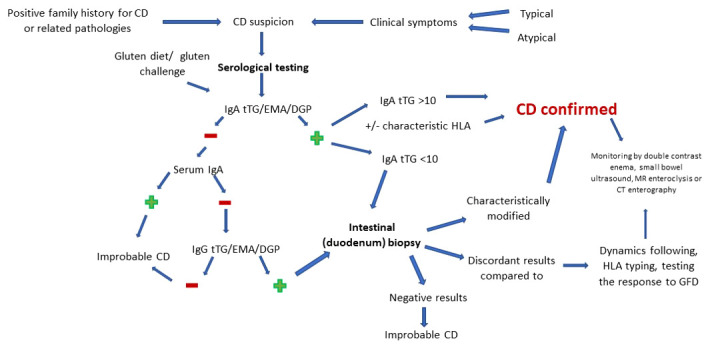
CD diagnostic scheme.

Since most diagnostic variants are subject to bias, there may be false negative or false positive variations in interpretation in certain situations, **Figure 1** details, in accordance with those previously presented, the optimal sequence to follow in order to obtain the most accurate diagnosis of CD.

## 5 Atypical forms of celiac disease

Comparing the classical form of presentation among young children, with the non-classical one encountered more frequently with advancing age, we conclude that, as researched and common in practice as this pathology has become in recent years, the more unclear it is for us still the whole range of manifestations that can accompany it. We can therefore say, without exaggerating, that the atypical form of CD is beginning to gain more and more prominence both in its description and in diagnosis (60, 61). Analyzing a group of 78 patients, Iwańczak B. et al. concludes that the prevalence of atypical celiac disease closely followed the classic form of the pathology. The dominant symptomatology was represented by recurrent abdominal pain, failure to thrive, short stature, anemia, altered liver enzymes, food allergy, and associated thyroid disorders (62). We can add to this long list of chameleon-like clinical manifestations, according to Cooney MJ. et al., intermittent, noncyclic fever, of unknown origin. The observation was raised in the case of a 16-year-old girl with serological and histological confirmed CD later. At the initiation of the GFD, the clinical symptoms and the changes in the paraclinical indices remitted (63). The literature records other accompanying pathologies, which later proved to mask celiac disease. Among these we note duodenal ulcer, recurrent (pseudocystic) pancreatitis, splenomegaly (important to differentiate from a lymphoproliferative disorder), urolithiasis, severe thrombocytosis, central retinal vein occlusion or Henoch-Schoenlein purpura (64–70). Regarding the variation in the level of tTG antibodies between the two types of the disease, Aleksandra B. et al. exclude this hypothesis based on a retrospective study (71). These represent only a part of the polymorphism characteristic of CD. Thus, the most accurate outline of the systemic manifestations that can accompany celiac disease is vital in differentiating it from symptomatically similar conditions. Among these we note anorexia nervosa, autoimmune enteropathy/HIV, infectious/eosinophilic gastroenteritis, enteritis, lactose/soy intolerance, bacterial superinfection, Crohn’s disease, hypogammaglobulinemia, Whipple’s disease or Zollinger- Ellison syndrome (72). Finally, to make the distinction between the two forms of celiac disease (typical versus atypical), **Table 1** compares their main clinical features.

**Table 1:** Clinical variations characteristic of typical versus atypical celiac disease.

Type of the condition	Typical celiac disease	Atypical celiac disease
Clinical manifestations	diarrhea with steatorrhea anorexia vomiting flatulence abdominal discomfort growth retardation severe malnutrition nutritional deficiencies anemia delay in the onset of puberty	small stature skin-mucosal manifestations osteoarticular, dental or skin appendages (hair) damage endocrinopathies hematological or neuro-psychic disorders increased risk of developing autoimmunity recurrent abdominal pain altered liver enzymes food allergy intermittent, noncyclic fever, of unknown origin accompanying pathologies: duodenal ulcer, recurrent (pseudocystic) pancreatitis, splenomegaly (important to differentiate from a lymphoproliferative disorder), urolithiasis, severe thrombocytosis, central retinal vein occlusion or Henoch-Schoenlein purpura

Currently, given the multitude of studies in the field, the heterogeneous presentation of celiac disease should no longer represent an obstacle in the timely identification of the diagnosis. In order to facilitate this process, we detail below the main atypical forms of CD manifestation, summarized in **Table 2** (adapted from subsection 5). At the same time, we outline the possible causes that determine their appearance, but also means of therapeutic approach where they can improve the prognosis or the quality of life.

**Table 2:** Atypical manifestations of CD depending on the location of the damage (adapted from subsection 5).

Type	Associated manifestations
Constitutional features	− Stature deficit − Excess weight
Biological disorders	− Anemia (iron deficiency, inflammatory, aplastic thalassemia) − Elevated transaminases − Hypoalbuminemia − Coagulopathy − Severe thrombocytosis − Anti-phospholipid antibodies − Hydro-electrolytic imbalances
Systemic disorders	General: − Non-alcoholic steatohepatitis − Hypotension − Epistaxis/ecchymoses/hemorrhages − Intermittent, non-cyclical fever − Duodenal ulcer − Recurrent (pseudocystic) pancreatitis − Splenomegaly − Central retinal vein occlusion − Henoch-Schoenlein purpura − Intestinal intussusception
	Cutaneous and mucous membranes: − Dermatitis herpetiformis − Atopic dermatitis − Pemphigus − Linear IgA bullous dermatosis − Chronic urticaria − Psoriasis − Vitiligo − Rosacea − Pellagra − Hereditary angioneurotic edema − Vasculitis − Nodosum/necrolytic migratory erythema − Behçet’s disease − Dermatomyositis − Oral lichen planus − Hypertrichosis − Damage to the dentition (delayed tooth eruption, mineralization defects, dental caries) − Lesions of the oral mucosa (recurrent canker sores, ulcers, erythema, cheilitis, atrophic glossitis) − Dryness and burning sensation of the tongue
	Renal: − Renal diabetes − IgA nephropathy − Glomerulonephritis − Renal damage with minimal damage − Focal and segmental glomerulosclerosis − Nephrotic syndrome − Urolithiasis − Urinary infection − Distal tubular renal acidosis
	Bone and joint damage: − Hypomotility − Proximal muscle weakness − Arthralgia − Osteoporosis − Osteopenia − Increased risk of fracture
	Neuropsychiatric disorders: − Gluten ataxia Peripheral neuropathy (Guillain-Barrè syndrome) − Headache − Migraine − Epilepsy − Depression − Anxiety − Cognitive-behavioral disorders (dementia, memory disorders, acalculia, lack of attention, executive or spatial orientation deficits)
	Reproductive: − Delayed puberty − Irregularity of menstruation − Amenorrhea − Early menopause − Risk of spontaneous abortion − Children with low birth weight − Reduced breastfeeding capacity − Stillborn children
Other associated diseases	Autoimmunities: − Diabetes mellitus − Hashimoto’s thyroiditis − Graves’ disease − Addison’s disease (adrenal insufficiency) − Systemic lupus erythematosus − Secondary hyperparathyroidism − Ovarian insufficiency − Pituitary disorders − Autoimmune hemolytic anemia − Autoimmune hepatitis
	Notable: − Down syndrome − Asthma

### 5.1 Deficient development curve

Children’s height is evaluated by comparing it with the anthropometric standards characteristic of each age group. Thus, the child is classified as small stature when his height falls below two standard deviations relative to the age-specific percentile. Disturbance of growth in height requires an extensive assessment carried out mainly with the aim of early diagnosis of the etiology and the initiation of appropriate treatment to be able to recover the deficit (73). Isolated short stature has been reported globally in 10% to 47.5% of celiac patients. The data are also supported by a study conducted on 104 Iranian children, who noted a prevalence of celiac disease of 33.6% among children with stature deficiency. The authors also emphasize the important correlation between growth retardation and positivity of CD-specific serological screening tests and histological morphology (9, 74). Similar findings were reported in Saudi Arabia, Assiri AM. et al. supporting the link between small stature and CD, although their research objectified a prevalence of only 10.9% of the total number of included patients (75). Currently, it is considered that up to 1/3 of children with short stature may have associated CD, independent of the age and gender of the patients, but this is controversial (76, 77). In order to practically exemplify the usefulness of knowing the connection between the two entities, we bring into discussion the case of a 4-year-old girl, who was in the records since the age of 4 months for low growth rate, accompanied by malnutrition, without systemic damage (gastrointestinal, renal or endocrinological). In dynamics, serological values, HLA genotyping and histological examination finally confirmed the diagnosis. With the initiation of the GFD, the growth deficit experienced a significant improvement (78). Similar effects were observed by Soliman AT. et al. two years after starting the GFD, both in terms of waist and body mass index (79). As a result, we can conclude that the main factor that imprints the stature deficit among children with celiac disease is represented by dietary gluten. Extrapolating the findings, we can state that the wide range of short stature prevalence in CD, variable from a demographic point of view, can be largely attributed to the variability of the gluten concentration to which affected children are exposed.

Although this can be assimilated as a consequence of malnutrition, its detailed investigation is important especially among patients whose puberty has already been triggered. The literature attests to a height compensation 24 months after the initiation of GFD. However, results are poorly represented post-puberty. Pre-puberty, however, it is important to consider the differential diagnoses of small stature (e.g., growth hormone deficiency, inflammatory bowel disease, Turner syndrome). These can be solved by appropriate treatment, the mistake of assigning them exclusively CD can significantly decrease the chances of reaching an optimal final size (44). Newer theories regarding small stature associated with CD aim at the involvement of a dysfunction in the balance of insulin-like growth hormone (IGF1) and ghrelin. CD therefore appears to manifest itself by decreasing their levels, together with those of binding proteins 1 and 3. The theories underlying this dysfunction aim either at an association of autoimmunity directed against the pituitary gland, or at a disturbance supported by systemic inflammation (80). Giovenale D. et al. recommends, based on a large-scale retrospective study, the evaluation of serum GH levels among children who do not show an improvement in the growth curve despite GFD (81). Therefore, we can conclude based on the studies in the literature that CD and GH deficiency represent two key entities in pediatric practice, especially during the growing period. They can coexist, so the objectification of one should not stop the clinician from excluding the other. The optimal treatment in this case aims at both the supplementation with recombinant growth hormone and the therapy of the underlying gastrointestinal disorders (82, 83).

### 5.2 Excess weight

Although the body weight of patients with CD most often follows a decreasing trend, clearly attributed to nutritional and absorption deficiencies, the occurrence of weight gain or even obesity cannot be neglected. This manifestation can accompany the pathology from the beginning, or it can appear later in evolution. The physiological bases of this paradoxical clinical manifestation have tried to be explained by means of two theories. The “compensatory hypothesis” is defined as the increased absorption capacity of the distal intestine, a segment that undergoes a functional adaptation following duodenojejunal atrophy. Considering the fact that the intestinal compensatory surface increases with age, this theory is considered to be in agreement with the peculiarities of CD depending on the life stage in which the patient is. Another theory is that of gluten withdrawal. This presupposes the improvement of the intestinal absorption capacity due to the healing of the mucosa after initiation of GFD, as well as the inclination towards a diet with a high content of proteins and lipids, with a high content of sugars and low fiber among these patients (84). However, studies on adults do not attest to a direct correlation between the patient’s nutritional status and the variability of gastrointestinal symptoms (85).

According to the latter theory, Rodrigues M. et al. report a more significant increase in weight in the first two years of initiation of therapy. Similar results were obtained by Radlović N. et al., although they emphasize the absence of a significant difference between adherent and non-adherent patients to therapy (86, 87). The specialized literature records that excess weight in newly diagnosed patients is more frequently associated with abdominal pain as an accompanying manifestation. The importance of screening among children with excess weight, with recurrent headaches of unspecified etiology, as well as close relatives of patients with CD who show excessive growth of body mass, is emphasized (88, 89).

### 5.3 Disorders of biological constants

Anemia is one of the most common extra-intestinal manifestations associated with CD. This can be the meeting in various forms, from iron deficiency anemia to thalassemia, inflammatory anemia or other types, as observed by Sanseviero MT. et al. From their reports, it appears that the gender distribution is approximately 2-1 in favor of girls. Also, cases of aplastic anemia associated with CD have been reported. The causality underlying it can be multiple, starting from nutritional deficiencies of iron, folate, vitamin B12 (due to villous atrophy or iron blocking in stores), blood loss and reaching the consequences of chronic pro- inflammatory status. In addition to these, in the case of CD, the polymorphism of the apical divalent transporter (DMT1). In case of massive iron loss due to villous atrophy and decreased absorption capacity, it may become unable to compensate despite its tendency to overexpress. Depending on these, the characteristics of erythrocytes vary (volume, color).

Regarding the serum level of iron and ferritin, we note that they are low, the value of hepcidin is variable, being able to even reach an increase in some cases. Similar findings are observed in the case of IL-2, which also recorded an increase (90–96). Other studies attest to an increase in ferroportin, doubled by the decrease in hephaestin, among children with celiac disease, compared to healthy ones (97).

Knowing and balancing these deficiencies is important especially at the pediatric age, when hyposideremia can negatively influence neuropsychological development. A known fact regarding the CD-anemia relationship is the disruption of the effective response to supplementation with oral preparations based on iron. In this case, the correction of the anemia will either follow the natural evolution of the CD therapy, or it will be done with intravenous preparations, depending on its severity. This interrelation was also demonstrated by Shahriari M. et al. in a study that targeted 184 children and adolescents. The authors thus emphasize the importance of early screening for the complications of celiac disease and their prevention (98, 99). Regarding the course of the underlying disease, the influence of anemia on the therapeutic response of GFD remains under investigation. However, the objectification of anemia since the CD diagnosis coincided with a more severe serological and histological profile. Also, the ability to return to normal hemoglobin was decreased, they recorded decreased values compared to the non-anemic group even after a year of GFD (100, 101).

In addition to affecting hemoglobin and nutritional components, CD has also been shown to be associated with the disruption of liver parameters and function. The main theory underlying this link is the interaction between toxic substances and the liver parenchyma.

These occur mainly due to the alteration of intestinal permeability that allows their passage into the portal circulation, inducing inflammation with liver lesions. Besides this, the involvement of immune factors cannot be neglected either, an aspect certified by the objectification of liver deposits of anti-tTG antibodies (80, 102). Also, in agreement with what was stated previously, the initiation of GFD can predispose to weight gain, especially in the first years. This disruption of body balance, together with the change in intestinal permeability, have been shown to be correlated with the increased risk of developing non- alcoholic steatohepatitis/fatty liver and metabolic syndrome. The stated pathological processes are chronic, with slow evolution and severity proportional to the duration. The risk was higher in the first years after the diagnosis but persisting up to 15 years after that. At the same time, it was higher among men compared to women and among children (103–105). From a biological point of view, it seems that elevated levels of liver enzymes (transaminases) are more common among young children with CD, increasing with intestinal alteration. They show a correlation with specific celiac disease antibodies, their increased titers being recorded in cases of hypertransaminasemia (106).

Therefore, the hepatic manifestations of CD should not be neglected. Ignoring this correlation leads to vices of etiological attribution. Severe insufficiency can be reached in time, with the need for grafting in order to restore the homeostasis of the internal environment, consequences that could be avoided by excluding gluten at the right time (107–109). Other biological disorders include hypoalbuminemia and disturbances at different levels of the coagulation cascade, platelet abnormalities, the presence of anti-phospholipid antibodies or vitamin K deficiencies. All these disorders underlie the predisposition to generalized edema and hemorrhagic or thromboembolic events, characteristic of CD (110, 111). Cases of diffuse infiltration, hydro-electrolytic imbalances, hypotension, epistaxis, spontaneous ecchymoses and even intramuscular hemorrhage occurring in the context of coagulopathy or celiac crisis are recorded in adults and children (112–114).

### 5.4 Skin and skin appendages manifestations

We can say without exaggerating about the skin that it represents the map of the body. Thus, the relationship between the body and the skin is bidirectional. Both the disturbances of the internal environment are reflected at its level, and vice versa, the disturbance of its integrity determines consequences at the systemic level (115). Regarding the main mechanisms incriminated in the appearance of skin lesions in CD, it seems that they are represented by the generation of immunoglobulins, pro-inflammatory cytokines and circulating immune complexes in response to the reactions carried out in the intestinal submucosa (116). Many works have been written regarding the skin manifestations encountered in the atypical form of CD. Summarizing, Abenavoli L. et al. review the most important conditions that presented statistical significance based on the analysis of randomized studies, cohort studies, case series or systemic reviews. Among these we note vesicular diseases (pemphigus, dermatitis herpetiformis, bullous linear IgA dermatosis), urticaria, psoriasis, vitiligo, rosacea, pellagra, hereditary angioneurotic edema, atopic dermatitis, cutaneous vasculitis, nodosum/necrolytic migratory erythema or elevatum diutinum, Behçet’s disease, dermatomyositis, oral lichen planus or acquired hypertrichosis (117). Similar findings were presented by Turjanmaa E. et al. They also emphasized that the severity of the skin manifestations did not vary between the study groups (118). Also, these conditions can overlap, there are forms of urticaria herpetiform dermatitis described in the literature (119). In order not to distract attention from the frequently encountered skin manifestations, we choose to detail them in the following.

Dermatitis herpetiformis (also called centrifugal annular erythema) is characterized by a vesicular, itchy rash, distributed mainly on the elbows, knees, sacral region and buttocks. Occasionally, the upper back, abdomen, scalp or face can also be affected. The reasoning behind this preference for extensor surfaces resides in the fact that they are much more prone to injury/inflammation due to the bending action of the joint. At the same time, during flexion, periarticular capillaries and nerves are stretched. Definitive diagnosis requires a skin biopsy, followed by analysis of the fragment by direct immunofluorescence. Thus, the deposition of granular immunoglobulin A (IgA) in the papillary dermis (dermo-epidermal junction) is objectify. The long-term prognosis is encouraging, the treatment of the condition being based on the administration of topical clobetasol or dapsone (diamino-diphenyl sulfone) (80, 116, 120, 121). The differential diagnosis must be performed with centrifugal annular erythema. It is presented by lesions with a polycyclic outline, erythematous or vesicular, which extend towards the periphery. They may or may not be accompanied by itching. The evolution of GFD pot is marked by the remission of skin lesions, with minimal residual hyperpigmentation (122).

Chronic urticaria (lasting more than 6 weeks) is a skin manifestation known to be associated with autoimmune diseases. The underlying mechanism is largely attributed to the existence of autoantibodies against the high-affinity immunoglobulin E (IgE) receptor (FcϵRI). To this is added the existence of a fund of chronic systemic inflammation (123, 124). The relationship between the two manifestations is bidirectional, there is currently no unanimity regarding their sequence (125, 126). A particular case of its presentation was recorded in a boy aged 3 years and 8 months. He complained of hives and angioedema after exposure to low temperatures. The symptomatology was doubled by persistent iron deficiency anemia.

Investigations in dynamics have objectified CD with the help of serological and histological tests (127). Similar cases were also described later in the literature (128). In this case, in addition to GFD, preparations such as non-sedating/sedating antihistamines or oral steroids can be used. The latter must be used judiciously, by the allergist specialist, to avoid over- administration (129).

Rosacea is a chronic inflammatory condition manifested mainly in the central part of the face. The pathognomonic lesions are represented by persistent erythema, papules, pustules and telangiectasia. These may or may not be accompanied by eye involvement, oedema, burning/stinging or xerosis. Its pathophysiology brings together the disruption of epithelial barriers, genetic and environmental factors, immunological imbalances and neurovascular reactions. It shares common susceptibility loci with CD, having a more significant statistical association among females than males. In boys, the autoimmune disease that reached statistical relevance regarding the association with rosacea is rheumatoid arthritis (130–132).

The incidence rate of alopecia among newly diagnosed CD patients is estimated to be ~1%. Conversely, the prevalence of CD among children with alopecia is almost 50%. The importance of performing screening in the affected population is therefore explained. The gender ratio is in favor of girls, they more frequently present an association between alopecia and CD. The main pathogenic mechanism incriminated in its occurrence is the autoimmune one. In this sense, hair regeneration 1-2 years after GFD initiation attests to the hypothesis.

Depending on the type of lesions, the condition can be divided into alopecia areata, totalis and universalis (80, 133). The case reports attest to the correlation between the two entities. Also, the results of the gluten-free regimen on hair regeneration are reiterated (134, 135). To this, for an adjuvant purpose, we add the promising prospects of the Mediterranean diet, rich in protein and soy (136).

Affecting the components of the oral cavity (mucous membranes, dentition) can be identified as an accompanying manifestation of celiac disease. Chronic CD can be associated with delayed tooth eruption, mineralization disorders and dental caries, recurrent canker sores, lesions of the oral mucosa (ulcerations, erythema, cheilitis, atrophic glossitis), dryness and burning sensation of the tongue. The main mechanism incriminated is oral dysbiosis. The consequences are an oral smear rich in leukocytes, a change in the salivary volume and the structure of the tests. Affecting the enamel can involve the initial dentition or the permanent dentition. They are frequently arranged symmetrically, at the level of the four quadrants of the dental arch, having a double prevalence among the celiac population, compared to the general population. The main drawback lies in the lack of response to the exclusion regime in the case of permanent dentition, an effect that does not occur in the case of stomatitis (44, 137–143). Multiple studies in the recent medical literature attest to the correlation (144–146).

Recurrent aphthous stomatitis specific to celiac disease is defined as the presence of multiple lesions on the non-keratinized oral mucosa, usually occurring in childhood or adolescence.

Broadly speaking, it is found in 4% to 41% of CD patients. The appearance is round or ovoid, bordered by an erythematous background, with a yellow or gray base. Their presenceinterferes especially with speech, mastication and swallowing, decreasing the quality of life due to pain. Regarding disturbing factors, it seems that HLA DQB1 has a protective role, while family predisposition, local trauma, stress, hormonal variations, nutritional deficiencies, food hypersensitivity or immune changes leave a negative impression on the patient’s chances of developing the disease (147, 148). CD screening among children with recurrent aphthous stomatitis and nutritional deficiencies is vital, the incidence of the pathology being significantly increased compared to the general population (149). GFD together with the local, individualized treatment of the lesions has demonstrated beneficial results in terms of restoring the oral health of patients with CD. The exception is found in what concerns the damage to the dental enamel (150).

### 5.5 Systemic damage

Atypical celiac disease at the onset can involve various organic manifestations. Among these, we highlight intestinal intussusception. Although its clear frequency could not yet be established, the association between the two pathologies is certified by numerous reported cases (151–154). It is noted that the risk factors associated with this are age and low weight, diarrhea at presentation, abdominal distension, rickets, low serum albumin, severe villous atrophy and refeeding syndrome (155, 156). The risks in the dynamics are represented by the recurrence of symptoms, intestinal obstruction and acute surgical abdomen. The differential diagnosis must be made with tuberculosis and intestinal lymphoma. Since the manifestation is often transient, expectant management and the initiation of GFD are preferred over surgical resolution (157, 158). In conclusion, for an optimal approach to intestinal intussusception, especially in small children, it is recommended to perform CD screening even in the absence of nutritional deficits (159).

Intestinal disorders that accompany CD can represent a cause of kidney damage, mainly through the intestine-kidney axis. Thus, celiac disease can predispose and precipitate the appearance of conditions such as renal diabetes, IgA nephropathy (IgAN), glomerulonephritis, renal damage with minimal damage, focal and segmental glomerulosclerosis, nephrotic syndrome, urolithiasis, urinary infection or distal tubular renal acidosis (160, 161). Also, subclinical CD can be associated with hyperoxaluria and a dynamic evolution towards deterioration of renal function (162). By far the most particular form of kidney damage in CD that can exemplify the gut-kidney correlation is IgAN. A similar effect in which dysbiosis is blamed can also be observed in chronic kidney disease. Recent studies have demonstrated its genome-wide association with immune-mediated inflammatory bowel diseases, intestinal barrier functionality, and response to intestinal pathogens. This overlap has a negative impact on the prognosis (163–165). A peculiarity of the renal samples collected from celiac patients is represented by the objectification of IgA-tTG deposits only in the case of those who do not follow a gluten exclusion diet (166). In conclusion, Pérez- Sáez MJ. et al. attests to the beneficial impact played by GFD in the biological evolution of children with kidney damage (167).

The characteristic symptomatology of the osteoarticular apparatus is intensely evoked mainly in childhood. Its causality can be dictated both by physiological processes specific to the period, and by disturbances in the pathological sphere. Regarding the subject of the study, we discovered causal associations between CD and joint hypomotility, proximal muscle weakness, arthralgias, osteoporosis, osteopenia and increased fracture risk. All theseassociated diseases are included under the umbrella of the term “metabolic bone disease” (168–170). As the name indicates, metabolic bone disease has as its main substrate malnutrition following the alteration of intestinal absorption. Added to this are the consequences of the chronic inflammatory state. Certainly, for a proper approach, the exclusion of food gluten is not enough. Although it showed promising results, the GFD must be accompanied by supplementing the deficiencies (168). GFD led to an increase in vitamin D levels, improvement in bone mass content and bone mineral density, although the period required for its normalization cannot yet be estimated. However, its role in bone health cannot be neglected (171–173).

The main neurological conditions particularly associated with CD are gluten ataxia, peripheral neuropathy (Guillain-Barrè syndrome), headache, epilepsy, depression, anxiety and cognitive-behavioral disorders. The latter include various forms of dementia (Alzheimer’s, vascular, frontotemporal), memory disorders, acalculia, lack of attention and deficits in execution or spatial orientation. Post-mortem examination in gluten ataxia objectifies as a distinctive sign the uneven loss of Purkinje cells in the cerebellar cortex.

Migraine is another manifestation possibly associated with celiac disease, the pathognomonic sign of the coexistence of the two entities being occipital and parieto-occipital calcifications. The hypotheses regarding the mechanism by which the central nervous system is affected are multiple. These can be briefly classified as cerebral hypoperfusion (due to perivascular inflammation) or gluten-mediated mechanisms. The latter include cross-reaction of autoantibodies, deposition of immune complexes, direct toxicity or disturbances of the gut- microbiota-brain axis (147, 174–178). Predisposing factors for neurological manifestations are female sex, mild histopathological form and HLA-DQ2 heterozygosity (179).

Bashiri H. et al. records a prevalence of approximately 6% of celiac disease among children with epilepsy. This encourages through their study the GFD approach, noting promising results in the control of convulsive seizures (180). Although the prevalence of CD did not reach a statistical significance among children with autism spectrum disorders, compared to the general population, Prosperi M. et al. underlines the need for screening in this group of patients. The considerations on which they are based are given by the identification of some patients with asymptomatic CD in the target group, but especially by the frequency of the atypical clinical picture in this age group (181). In addition, Özbudak P. et al. describe the celiac disease-catatonia association, by exemplifying the case of a 15-year-old girl.

Characteristic manifestations include stupor, waxy flexibility, and muteness lasting more than 1 hour (182). Also, although the case concerns a man past the age of adolescence, we want to mention the possibility of the onset of CD through neurological symptoms specific to amyotrophic lateral sclerosis. In this situation, the elimination of gluten for a period of 4 months coincided with the stopping of the evolution of the neurological symptoms and the normalization of the gastrointestinal ones (183). Recent studies also attest to the association at the genomic level of the two conditions (184). In conclusion, the differential diagnosis of subclinical or clinical neurological manifestations in children must include CD. The benefits aim at both the prevention of progressive deterioration in adult life, as well as the ability to exploit the protective effects of GFD in evolution (185).

Puberty usually occurs around the age of 11 for girls and 12 for boys. The evolution of secondary sexual characters is monitored using the Tanner classification. The differential diagnosis must include investigations such as the assessment of bone age, based on growthnuclei. In the context of CD, the delay in the onset of puberty is attributed to malabsorption and the delay in maturation of the hypothalamic-pituitary-gonadal axis. If in girls this manifests itself through delayed menarche, in boys we observe a mode suggestive of androgen resistance (reduced serum level of dihydrotestosterone and increased by luteinizing hormone). Therefore, if we are faced with delayed puberty, it is necessary to perform the screening for CD and initiate the GFD. The lack of improvement after 1-2 years of the regimen requires the patient to be referred to endocrinology for the exclusion/objectification and treatment of coexisting conditions (44, 186–188). Bayrak NA. et al. attests the correlation between Tanner stages, GFD adherence, transferrin saturation, total iron binding capacity and vitamin D (189).

In evolution, untreated CD can lead to reproductive disorders among girls past the age of adolescence. These are manifested by irregular menstruation, amenorrhea, early menopause, risk of spontaneous abortion, children with low birth weight, reduced breastfeeding capacity or children stillborn. The severity of the consequences is directly proportional to the severity of malabsorption. They fade and even go away with GFD adherence (190, 191).

Consequently, CD screening among women with unexplained infertility is important, Remes- Troche JM. et al. noting a positivity rate of at least one specific marker for them of approximately 4.6% (192). Affecting male fertility was not observed (193).

### 5.6 Association of autoimmune diseases

It is known in the medical world that autoimmune diseases can form familiar aggregates. However, the possible association between multiple autoimmunities found in the same patient should not be neglected either. In this sense, we will present in the following the autoimmune associations that can camouflage celiac disease.

Broadly speaking, specialized literature correlates CD with endocrinopathies such as diabetes, Hashimoto’s thyroiditis, Graves’ disease, Addison’s disease (adrenal insufficiency), secondary hyperparathyroidism, ovarian insufficiency or pituitary disorders (resulting in hypogonadism, prolactin level imbalances) (194, 195). Autoimmune hemolytic anemia or autoimmune hepatitis can also be added to this list (196). By studying a group of 228 patients, Varol Fİ. et col. notes the absence of predictive factors that can anticipate the risk of association of CD with other glandular autoimmunities. They analyzed age, sex, symptoms, tTG level, HLA haplotype and histopathological stage. They did not obtain statistical results except in terms of age (higher in the case of association) (197).

Exceptionally, the specialized literature records associations of CD with Down’s syndrome or asthma (198–200). In this case, screening is encouraged, as only intestinal histological changes can be detected, without immunological disturbances or CD specific markers (201). The consequences are among the most diverse, being able to include sideroblastic anemia resistant to iron treatment. This subsided when GFD was introduced into the therapeutic regimen (202). The pathogenic bases of the association are most likely aimed at immune and microbial imbalances, common to the three pathologies (203, 204). Similar implications can be observed in the case of the association CD - systemic lupus erythematosus, a condition in the etiology of which we also find dysbiosis (205). Therefore, a number of autoimmunities can overlap in some situations, being included in the multiple autoimmune syndrome (MAS). MAS is divided into three types, depending on the coexisting pathologies. In this sense, Boccuti V. et al. presents the case of a known boy with CD atypical form in the antecedents(psychiatric symptoms at the age of 18). He was also diagnosed with Hashimoto’s thyroiditis and systemic lupus erythematosus. The current association is included in MAS 3 (206).

Unlike the previous examples, where the connection with CD was explained from the perspective of similar pathogenesis, the overlap of CD - type 1 diabetes mellitus (T1DM) most likely refers to the common genetic predisposition (207). The prevalence estimated by Goh C. et al. it is approximately 6% if we refer to the positive serological testing for CD and 4% regarding the results of the characteristic jejunal biopsy (208). More recently, Joshi R. et al. notes the prevalence of CD in the group of those with diabetes as being 15%, with a positivity rate of the biopsy of 7% and a characteristic symptomatology that affects 1/3 of the patients at the time of screening. Also, they place the T1DM - CD correlation in second place, after that between T1DM and autoimmune thyroiditis (209). Furthermore, Singh P. et al. emphasizes that the coexistence of the two conditions increases the patients’ risk of developing hypothyroidism and small stature (210). Height retardation has not been shown to be correlated with gender, but it is dependent on the time of CD diagnosis (more important at younger ages) and following a GFD (211). Worthy of mention in this context is also the result of the study conducted by Delvecchio M. et al., who demonstrated an alteration of the absorption capacity of iodine among patients with CD, compared to the general population.

Also, this seems to be only partially corrected by the initiation of GFD (212). Finally, Bourhanbour AD. et al. discuss the prevalence of the CD-T1DM association in different areas of the world, by comparison with the Moroccan population. It also highlights the importance of CD screening in susceptible patients, especially due to the long-term risk of lymphoma or adenocarcinoma (213).

## 6 General therapeutic lines

The therapeutic gold standard of CD, respectively GFD, can be considered a necessary evil. In this context, some patients confess that they feel the burden of the gluten exclusion diet more acutely than that of the treatment for type 1 diabetes, intestinal or heart diseases. The exception was represented by patients with kidney disease in the dialysis stage, who felt the burden of the underlying disease more strongly. For this reason, current research focuses on the development of adjuvant therapeutic substances, intended to allow the inclusion of dietary gluten to be tolerated without triggering clinical symptoms and paraclinical manifestations. In this regard, we discuss prolyl endopeptidases, NKG2D antagonists, R-spondin-1, IL-15 blockers, glutenases (e.g., latiglutenase), the tight junction regulator (e.g., larazotide), nanoparticles that induce tolerance to gliadin, polysulfonated synthetic polymer (hydroxyethyl methacrylate-costyrene sulfonate), anti-gliadin antibodies from egg yolk and various immunoprophylaxis variants. However, their effectiveness remains controversial and open to future research (3, 47, 214–216). A benefit can be felt by patients when adding quinoa flour and malt to the diet. The results reside in the nutritional enrichment and increased food variability offered by the two components (217). Another adjuvant therapeutic approach is represented by the administration of steroids in recently diagnosed disease. However, this shows modest paraclinical results (218). To these is added the treatment of coexisting conditions and the supplementation of the main nutritional deficiencies of iron, calcium, magnesium, potassium, vitamin B12, A, D, K, folic acid or proteins (2).

Once CD is diagnosed and therapy initiated, a key point of management is the follow-up of adherence and its results. In this sense, it is important for the practitioner to know and avoidthe administration of medicines that contain traces of gluten in their composition.

Summarizing, we retain among its various preparations based on β-alanine, Acebutolol, Adenosine phosphate, Sulfadiazine, Cefaclor, Allopurinol, Phenobarbital, Trihexyphenidyl hydrochloride, Probenecid, Ketoprofen, Cholecalciferol, Ethambutol, Silymarin, Methotrexate and the list can go on (48). Of course, this specificity with possible implications for CD is dependent on the excipients and not the active substance. However, an alarm signal must be raised to raise awareness of the possible implications of some drugs commonly used in clinical practice, when their method of preparation is not fully understood by the clinician. The benefits of GFD regarding the reduction of celiac disease morbidity were most of the time indisputable, both for symptomatic and asymptomatic patients (219–221). The literature also notes superior results of GFD among children, compared to adults (222). Regarding the variability of the response to GFD, there is no data in the literature, to our knowledge, that attests different results in terms of efficiency between the typical and the atypical form. The distinction resides mainly in the manifestations that it diminishes/counteracts. Respectively, in the typical form of the disease, the aim is to obtain a histopathological remission at the level of the affected intestinal epithelium by using GFD. In contrast, taking into account the fact that extra-intestinal manifestations predominate in the atypical form, the desired in this situation is also the prevention of potential complications of the disease. Therefore, we can conclude that, while in classic CD GFD has mainly a therapeutic role, in the atypical form of CD it also plays a role in prevention. In both forms, the effectiveness of the GFD is rather influenced by the receptivity of the patients depending on their age (children show better results than patients whose therapy started at older ages) and the amount of gluten ingested “accidentally” (desirably below 10 mg/day). At the opposite pole, in the form of refractory celiac disease, we are mostly talking about a resistance to a GFD correctly followed and maintained for at least 12 months. This can be either primary or secondary (relapse after apparent results induced by GFD) (9, 72, 223). In this situation, GFD plays rather an adjuvant role, additional to nutritional therapy, pharmacological and surgical measures, rather than the main therapeutic one. It is noted that GFD reduces overall morbidity and mortality in CD. However, GFD alone may be an effective maintenance therapy in exceptional cases. Elemental diet showed promising results in a small heterogeneous group of patients with refractory enteropathy without clonal intraepithelial lymphocyte phenotype (refractory CD type 1) (42). Probiotics can represent adjunctive therapeutic means in the case of residual symptoms despite adherence to the GFD. For this purpose, preferably use preparations based on *Bifidobacterium* and *Lactobacillus*. The effects are marked by an increase in the *Firmicutes/Bacteroides* ratio and the abundance of *Actinobacteria*. To these is added the decrease of pro-inflammatory factors, acetic acid and total short-chain fatty acids, in parallel with the restoration of the microbiome (37). This clarification reinforces the conviction regarding the importance of early diagnosis and treatment of affected children, with the main aim of limiting the medium and long-term consequences.

In addition to GFD, adjuvant nutritional and pharmacological therapy, we also find an active lifestyle. In this sense, Nestares T. et al. argue that spending greater time in vigorous physical activity was associated with higher lean mass and bone mineral density, regardless of the time they followed a GFD (224).

## 7 Conclusions

The current work is focused on the atypical, non-pathognomonic phenomena encountered in celiac disease. We tried to expose in a clear, concise and objective manner the main signs, symptoms or conditions that can be associated with CD. The subject is a topical one, mainly because of the objectification of the increase in the diagnosis rate of celiac disease at the global level. Added to this is the ease with which most of the imbalances correlated from a causal or associative perspective with CD can find their solution with the initiation and maintenance of a GFD. Especially in late childhood, adolescence and even later in adult life, the classic form of the pathology is only the tip of the iceberg. At its base there are numerous constitutional, biological and systemic disturbances whose exposure were made with the aim of emphasizing the importance of screening in risk groups. This awareness is vital because, compared to other diseases whose methods of diagnosis and treatment may still encounter cognitive biases, CD is well described from this point of view. The gold standard is represented by the GFD, doubled by adequate nutritional supplementation.

The therapeutic target is mainly maintaining the metabolic balance in balance given its role in the child’s growth and development. There is currently no approved pharmacological treatment. In conclusion, the child with CD requires the formation around the disease of a multidisciplinary team, adequately trained in the knowledge, diagnosis and rapid countermeasures of the main associated symptoms. The period until the intervention dictates the long-term prognosis. However, it is not recommended to initiate a gluten exclusion regimen without clearly objectifying the pathology in order not to precipitate further consequences. Finally, we would like to draw attention to the wide range of pathologies entangled with CD, considering it necessary to deepen further research aimed at the physio- pathological bases of the interconnection. The main purpose of this initiative is the possible prevention of associated comorbidities (e.g., skin, kidney, brain damage by modulating the intestinal microbiota) and avoiding the exacerbation of the already existing ones.

## Author contributions

VL: Conceptualization, Methodology, Writing – original draft. MS: Investigation, Software, Writing – original draft. EJ: Investigation, Visualization, Writing – original draft. IS: Validation, Writing – review & editing. II: Conceptualization, Visualization, Writing – original draft. SR: Investigation, Software, Writing – original draft. VM: Software, Validation, Writing – review & editing. AN: Validation, Writing – review & editing. CD: Validation, Writing – review & editing. DS: Validation, Writing – review & editing. AK: Validation, Writing – review & editing. AL: Investigation, Methodology, Writing – original draft. AM: Writing – original draft, Writing – review & editing.
